# Ocrelizumab Depletes CD20^+^ T Cells in Multiple Sclerosis Patients

**DOI:** 10.3390/cells8010012

**Published:** 2018-12-28

**Authors:** Stefan Gingele, Thais Langer Jacobus, Franz Felix Konen, Martin W. Hümmert, Kurt-Wolfram Sühs, Philipp Schwenkenbecher, Jonas Ahlbrecht, Nora Möhn, Lars H. Müschen, Lena Bönig, Sascha Alvermann, Reinhold E. Schmidt, Martin Stangel, Roland Jacobs, Thomas Skripuletz

**Affiliations:** 1 Department of Neurology, Hannover Medical School, D-30625 Hannover, Germany; gingele.stefan@mh-hannover.de (S.G.); Konen.felix@mh-hannover.de (F.F.K.); Huemmert.Martin@mh-hannover.de (M.W.H.); Suehs.Kurt-Wolfram@mh-hannover.de (K.-W.S.); Schwenkenbecher.philipp@mh-hannover.de (P.S.); Ahlbrecht.jonas@mh-hannover.de (J.A.); Moehn.nora@mh-hannover.de (N.M.); Mueschen.Lars@mh-hannover.de (L.H.M.); Boenig.Lena@mh-hannover.de (L.B.); Alvermann.Sascha@mh-hannover.de (S.A.); Stangel.Martin@mh-hannover.de (M.S.); 2 Department of Clinical Immunology & Rheumatology, Hannover Medical School, D-30625 Hannover, Germany; Langerjacobus.thais@mh-hannover.de (T.L.J.); Schmidt.Reinhold.Ernst@mh-hannover.de (R.E.S.); Jacobs.Roland@mh-hannover.de (R.J.)

**Keywords:** Ocrelizumab, CD3^+^CD20^+^, CD20, T cells, B cells, multiple sclerosis, RMS, PPMS

## Abstract

Ocrelizumab, a humanized monoclonal anti-CD20 antibody, has shown pronounced effects in reduction of disease activity in multiple sclerosis (MS) patients and has recently been approved for the treatment of patients with relapsing MS (RMS) and primary progressive MS (PPMS). CD20 is mainly expressed by B cells, but a subset of T cells (CD3^+^CD20^+^ T cells) also expresses CD20, and these CD20^+^ T cells are known to be a highly activated cell population. The blood of MS patients was analyzed with multicolor flow cytometry before and two weeks after treatment with ocrelizumab regarding the phenotype of peripheral blood mononuclear cells. CD20-expressing CD3^+^ T cells were found in blood samples of all MS patients, accounted for 2.4% of CD45^+^ lymphocytes, and constituted a significant proportion (18.4%) of all CD20^+^ cells. CD3^+^CD20^+^ T cells and CD19^+^CD20^+^ B cells were effectively depleted two weeks after a single administration of 300 mg ocrelizumab. Our results demonstrate that treatment with ocrelizumab does not exclusively target B cells, but also CD20^+^ T cells, which account for a substantial amount of CD20-expressing cells. Thus, we speculate that the efficacy of ocrelizumab might also be mediated by the depletion of CD20-expressing T cells.

## 1. Introduction

Multiple sclerosis (MS) is a chronic inflammatory demyelinating disease of the central nervous system (CNS), which has long been considered to be primarily mediated by autoreactive T cells [1]. The compelling effectiveness of anti-CD20–directed therapies in reducing disease activity in MS patients has challenged this concept in recent years and put B cells and their role in the pathogenesis of MS into focus [2]. Phase I and II clinical trials with rituximab, a chimeric monoclonal anti-CD20 antibody, showed rapid and pronounced reduction of gadolinium-enhancing lesions and relapses in MS patients [3,4]. Following these promising results, ocrelizumab, a humanized monoclonal anti-CD20 antibody, was recently approved for the treatment of patients with relapsing MS (RMS) and primary progressive MS (PPMS), after pivotal Phase III studies demonstrated reduction of disease activity and progression [5,6].

CD20 is a cell-surface antigen mainly expressed by cells of the B cell lineage, encompassing B cell precursors and mature B cells but not hematopoetic stem cells and plasma cells [7,8]. Therefore, ocrelizumab is often considered to selectively deplete CD20-expressing B cells [6]. However, CD20 is also expressed on a small subset of CD3^+^ T cells. These CD20^+^ T cells display a distinct phenotype compared to the majority of CD20^−^ T cells, with an increased percentage of CD8^+^ and CD45RO^+^ cells [9]. CD20^+^ T cells represent a highly activated cell population, characterized by enhanced production of proinflammatory cytokines (e.g., tumor necrosis factor α (TNFα), interleukin-1β (IL-1β), and interleukin-17 (IL-17)) constitutively as well as upon activation [10,11,12]. These cells are found in blood, cerebrospinal fluid (CSF), and chronic brain lesions of MS patients [12,13]. CD20^+^ T cells have been shown to be effectively depleted by rituximab in rheumatoid arthritis and MS patients [10,14], and it has been speculated that targeting these CD20-expressing T cells might be an additional mechanism that could contribute to the clinical effectiveness of treatment with rituximab. However, ocrelizumab shows differences regarding binding to CD20 and exerting cytotoxicity [15], and it is not clear whether CD20-expressing T cells are depleted by ocrelizumab. Therefore, we aimed to investigate the impact of ocrelizumab treatment on CD20^+^ T cells.

## 2. Patients and Methods

### 2.1. Patients and Samples

Peripheral blood samples were collected from MS patients in the Department of Neurology at Hannover Medical School after written informed consent was provided. Blood was drawn directly before the first administration of ocrelizumab (dosage of 300 mg) and after 2 weeks, immediately before the second dose. Blood samples of 21 patients were investigated. Median age was 43 years, ranging from 22 to 65 years. Male-to-female ratio was 1:1.6. Patients had a diagnosis of either RMS (*n* = 17), with a median disease duration of 14.6 years, or PPMS (*n* = 4), with a median disease duration of 5.6 years. Detailed patient characteristics are given in Supplementary Table S1.

### 2.2. Multicolor Flow Cytometry

Phenotyping of lymphocytes was performed by incubating 200 µL of freshly drawn whole blood with antibodies in 5 mL tubes according to the manufacturer’s recommendations. The following antibodies were used for this study: CD45 APC-Cy, CD3 PE-Cy7, CD4 FITC, CD8 PB, CD19 BV 510, and CD20 PE (BD Biosciences, Heidelberg, Germany), CD56 BV 421 and TCR1PerCP Cy5.5. If not otherwise stated, antibodies were purchased from BioLegend (London, UK). Corresponding isotype-matched antibodies were used as controls. After 20 min of incubation, 2 mL of lysis solution from BioLegend was added to each tube, and 10 min later tubes were centrifuged (3 min at 400× *g*) and cells were washed 3 times with 2 mL of phosphate buffered saline supplemented with 0.1% bovine serum albumin (PBS/BSA) (3 min at 400x *g*). Finally, cells were resuspended in 250 µL of PBS/BSA and subjected to flow cytometry analysis on a FACS Canto II (Becton Dickinson, Heidelberg, Germany) by gating on CD45^+^ singlet lymphocytes (see Supplementary Figure S1) and acquiring 5 × 10^4^ to 1 × 10^5^ events per sample in the combined gate. Offline data analysis was performed by using FCS Express V6 software (De Novo, Glendale, CA, USA). Exact tSNE transformation was performed on a sample size of 5000 with 500 iteration steps and perplexity value of 30 after gating on T cells (CD3^+^), CD4^+^ T cells (CD3^+^CD4^+^), CD8^+^ T cells (CD3^+^CD8^+^), CD3^+^CD20^+^ T cells (CD3^+^CD20^+^), NK cells (CD3^−^CD56^+^), and B cells (CD19^+^) using the integrated tSNE function of FCS Express.

### 2.3. Statistical Analysis

All statistical analyses were performed using GraphPad Prism 8.0 (GraphPad Software, San Diego, CA, USA). Data are given as arithmetic mean ± standard error of the mean (SEM) and were further analyzed using the Wilcoxon matched-pairs test. Results were considered statistically significant at *p* ≤ 0.05 (*), *p* ≤ 0.01 (**), *p* ≤ 0.001 (***), and *p* ≤ 0.0001 (****) 

## 3. Results

### 3.1. CD20^+^ T Cells Constitute a Significant Proportion of CD20^+^ Cells in the Blood of MS Patients

**Figure 1:**
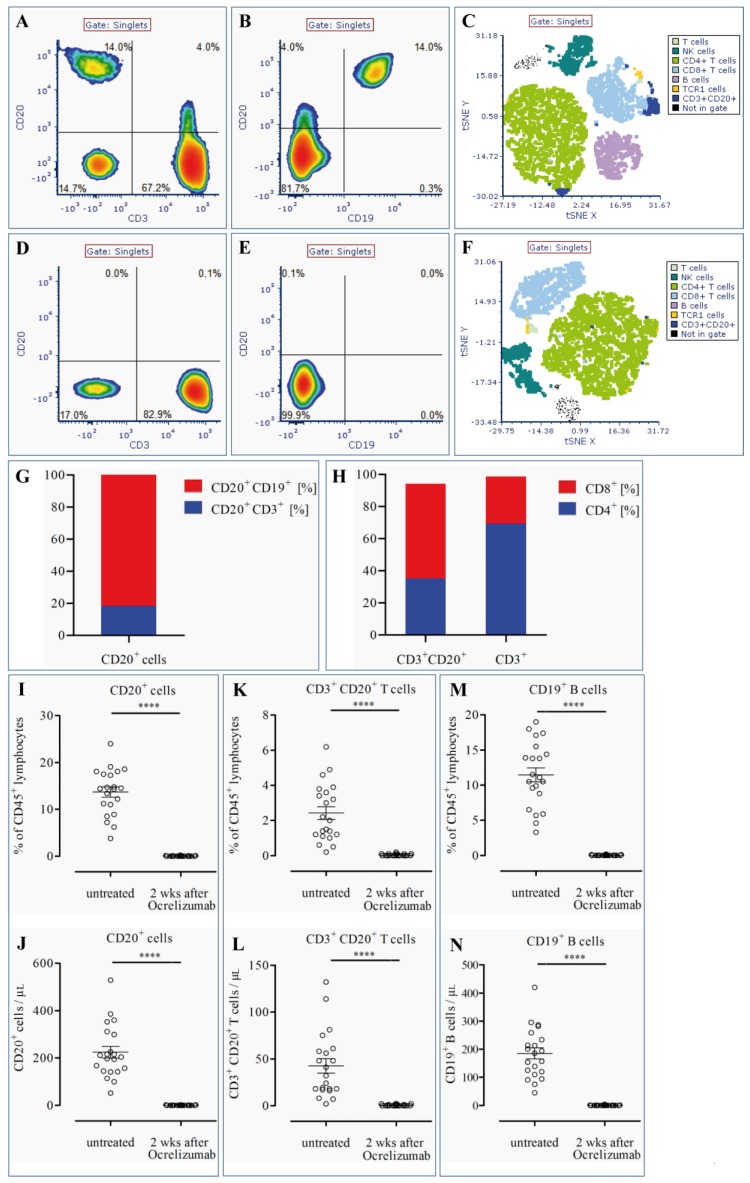
Detection of CD3^+^CD20^+^ T lymphocytes in peripheral blood of MS patients and depletion by ocrelizumab.

To identify lymphocyte cell distribution and phenotype, blood samples of MS patients were stained with a combination of eight antibodies and analyzed by flow cytometry. Exemplary plots of singlet cells before treatment with ocrelizumab (Figure 1A–C) demonstrate that CD3^+^CD20^+^ T cells represent a significant proportion of CD45^+^ lymphocytes in MS patients. CD3^+^CD20^+^ T cells were found in all patients and accounted for 2.4 ± 0.36% (mean ± SEM) of CD45^+^ lymphocytes (Figure 1K). In total numbers, the mean amount of CD3^+^CD20^+^ cells was 42.5 ± 7.7/µL in untreated MS patients (Figure 1L). Strikingly, CD20-expressing T cells constituted 18.4 ± 2.3% of all CD20^+^ cells (Figure 1G) with the remainder being CD19^+^ B cells (Figure 1B,G). Analysis of the co-expression pattern of CD20^+^ T cells showed that a higher proportion of CD3^+^CD20^+^ T cells co-expressed CD8 (58.9 ± 2.6%) compared to CD4 (35.1 ± 2.4%). In contrast, the entire CD3^+^ T cell population showed a lower percentage of CD3^+^CD8^+^ cells compared to CD3^+^CD4^+^ cells (29.2 ± 2.4% vs. 69.3 ± 2.4%; Figure 1H), being in line with the normal distribution of CD8- and CD4-positive T cells in peripheral blood. Only 1.8% ± 0.3 of CD4^+^ T cells were CD20^+^. The mean fluorescence intensity (MFI) of CD20 on CD4^+^ T cells was 1094 ± 70.13; 6.9% ± 1.0 of CD8^+^ T cells were CD20^+^ and the MFI of CD20 on CD8^+^ T cells was 1540 ± 111.8. These results demonstrate that predominantly CD8^+^ T cells express CD20. All of the CD19^+^ B cells were CD20^+^, and the MFI of CD20 on CD19^+^ B cells was 40,262 ± 3208. This finding confirms that CD20 expression on CD20^+^ T cells is considerably smaller than on B cells.

### 3.2. CD20^+^ T Cells Are Effectively Depleted by Ocrelizumab

Two weeks after the first administration of 300 mg ocrelizumab, blood samples of MS patients were again analyzed by multicolor flow cytometry to identify the effect of ocrelizumab on CD20-expressing lymphocytes. Illustrative flow cytometry plots (Figure 1D–F) show that CD3^+^CD20^+^ T cells as well as CD20^+^CD19^+^ B cells were rapidly and efficiently depleted from peripheral blood of MS patients after one dose of ocrelizumab. CD20-expressing cells, which amounted to 13.7 ± 1.1% (mean ± SEM) of the lymphocyte population and 224.9 ± 24.6 of absolute cell number/µL in MS patients before administration of ocrelizumab, were nearly completely depleted, with a frequency of 0.04 ± 0.01% and an absolute cell count of 0.57 ± 0.18/µL after treatment (*p* < 0.0001) (Figure 1I,J). Consistently, CD20-expressing T cells were also nearly fully diminished to 0.04 ± 0.01% of CD45^+^ lymphocytes and to a cell number of 0.57 ± 0.18/µL (*p* < 0.0001) (Figure 1K,L). In line with these findings, CD19^+^ B cells showed a nearly complete decrease two weeks after ocrelizumab treatment, to a frequency of 0.02 ± 0.01% of the lymphocyte population and a total cell count of 0.33 ± 0.19/µL (*p* < 0.0001) (Figure 1M,N), two weeks after administration of ocrelizumab.

Patients’ blood samples were stained with a combination of eight antibodies. Exemplary plots of singlet cells inside the CD45^+^ lymphocyte gate prior and two weeks after ocrelizumab treatment are shown in Figure 1A–C and D–F, respectively. Expression patterns of CD3 vs. CD20 (A, D) and CD19 vs. CD20 (B, E) are depicted. tSNE plots (C, F) exhibit the different lymphocyte populations listed in the inserted legends before (C) and 14 days after ocrelizumab-mediated B cell depletion (F). Mean cell distribution of CD20^+^ cells before treatment was between CD19^+^CD20^+^ B cells and CD3^+^CD20^+^ T cells (G). Coexpression patterns of CD4/CD8 in CD20^+^ T cells and the entire CD3^+^ T cell population are shown before treatment with ocrelizumab (H). Quantification of frequency of CD20^+^ (I, J), CD3^+^CD20^+^ (K, L), and CD19^+^ (M, N) cell populations is shown before and 14 days after administration of ocrelizumab. Dot plots show mean ± SEM with **** *p* < 0.0001.

## 4. Discussion

Although the expression of CD20 on a subset of CD3^+^ T cells was described more than 20 years ago [9], the expression of CD20 is often considered to be restricted to cells of the B cell lineage, and therefore anti-CD20–directed therapies are thought to specifically target B cells [6]. Our study clearly demonstrates that CD3^+^CD20^+^ T cells are commonly found in the peripheral blood of MS patients and represent a substantial proportion of CD20-expressing cells, accounting for 18.4% of all CD20^+^ cells. Thus, anti-CD20 antibody therapy should not be regarded as B cell–specific therapy.

Depletion of CD20^+^ T cells by anti-CD20–directed therapy was previously described for rituximab, a chimeric monoclonal anti-CD20 antibody, in rheumatoid arthritis and MS patients [10,14]. Ocrelizumab is the first monoclonal anti-CD20 antibody to be approved for the treatment of patients with RMS and PPMS. When compared to rituximab, ocrelizumab binds to a slightly different epitope and mediates its effects on CD20-expressing cells by greater antibody-dependent cellular cytotoxicity (ADCC) but lower complement-dependent cytotoxicity (CDC) activity [15]. Thus, it was not clear whether ocrelizumab might also deplete CD20^+^ T cells that express CD20 to a lower degree than B cells [9]. Our data reveal that CD20-expressing T cells together with CD19^+^CD20^+^ B cells are rapidly depleted already 14 days after administration of one 300 mg dose of ocrelizumab.

Since CD20 is perceived to be a B cell–specific marker, different functions of B cells, notably (auto-)antibody production, antigen presentation, and secretion of proinflammatory cytokines, have been suspected to account for the compelling effects of anti-CD20–directed therapies [3]. In contrast, CD3^+^CD20^+^ T cells are a highly activated subset of T cells that display increased expression of activation markers and production of proinflammatory cytokines (e.g., TNFα, IL-1β, or IL-17) that are suspected to be involved in the pathogenesis of MS, even in resting condition [10]. Treatments with the anti-CD20 antibodies rituximab and ocrelizumab result in a rapid and pronounced reduction of disease activity in MS patients after four weeks, as shown by a decrease of gadolinium-enhancing lesions [3,16].

In conclusion, these findings suggest that CD20^+^ T cells might play a pivotal role in the pathogenesis of MS, and we speculate that depletion of CD3^+^CD20^+^ cells by anti-CD20 monoclonal antibodies might contribute to the efficacy of anti-CD20 therapy in reduction of disease activity in patients with MS. However, it has to be kept in mind that our work represents a proof-of-concept study, therefore these results should be confirmed in further studies with larger cohorts of MS patients.

## Supplementary Materials

The following are available online at http://www.mdpi.com/2073-4409/8/1/12/s1: Figure S1: Gating strategy. Cells were gated on lymphocytes according to their FSC vs. SSC properties (**A**) further restricted to CD45^+^ cells (**B**) and by excluding doublets in a FSC-H vs FSC-A plot (**C**), Table S1: Patient characteristics.
