# Supplementary material for: Ocrelizumab Depletes CD20+ T Cells in Multiple Sclerosis Patients
Source: Cells. 2018 Dec 28;8(1):12. doi: 10.3390/cells8010012 (PMC6356421; doi:10.3390/cells8010012)
Supplement: Supplementary file 1 [file cells-08-00012-s001.pdf]

Supplementary

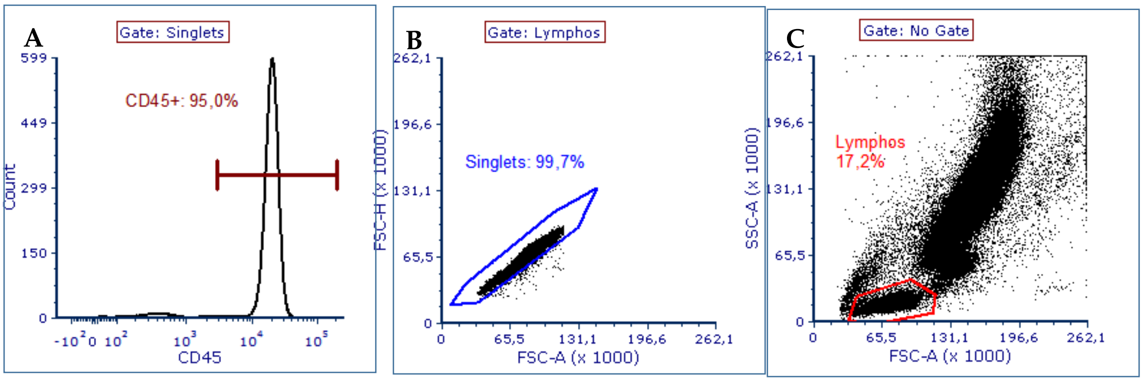

Figure S1. Gating strategy. Cells were gated on lymphocytes according to their FSC vs SSC properties (A) further restricted to CD45+ cells (B) and by excluding doublets in a FSC-H vs FSC-A plot (C).

Table S1. Patient characteristics.

Patient #	Male / Female	Age	Diagnosis	Last disease modifying therapy (DMT)	Interval between last DMT and ocrelizumab (months)	CD20+ T cells % of CD45+ lymphocytes
1	f	44 y	RMS	none	-	4.6 %
2	f	43 y	RMS	none	-	3.0 %
3	f	42 y	RMS	none	-	1.4 %
4	m	47 y	RMS	none	-	1.5 %
5	m	36 y	RMS	none	-	1.4 %
6	f	22 y	RMS	none	-	0.5 %
7	f	51 y	RMS	none	-	6.2 %
8	f	39 y	RMS	none	-	1.0 %
9	f	65 y	PPMS	none	-	3.6 %
10	m	26 y	PPMS	none	-	2.2 %
11	f	63 y	PPMS	none	-	3.4 %
12	m	54 y	PPMS	none	-	3.8 %
13	m	56 y	RMS	Glatirameracetat	11	4.9 %
14	f	48 y	RMS	Dimethylfumarat	8	0.2 %
15	m	52 y	RMS	Natalizumab	2	3.9 %
16	f	50 y	RMS	Interferon beta-1a	2	1.1 %
17	f	23 y	RMS	Teriflunomid	2	1.2 %
18	m	35 y	RMS	Dimethylfumarat	2	0.6 %
19	m	31 y	RMS	Natalizumab	2	3.2 %
20	f	28 y	RMS	Dimethylfumarat	2	1.2 %
21	f	31 y	RMS	Fingolimod	2	2.0 %
